# A Multimodal Adaptive Framework for Social Interaction with the MiRo-E Robot

**DOI:** 10.3390/s26041209

**Published:** 2026-02-12

**Authors:** Yufeng Yang, Pei Shan Yap, Sobanawartiny Wijeakumar, Aly Magassouba, Nikhil Deshpande

**Affiliations:** 1 School of Computer Science, University of Nottingham, Nottingham NG8 1BB, UKaly.magassouba@nottingham.ac.uk (A.M.); 2 School of Psychology, University of Nottingham, Nottingham NG7 2RD, UK; sobanawartiny.wijeakumar@nottingham.ac.uk

**Keywords:** human–robot interaction, multimodality, emotion expression, user experience evaluation, LLM-based adaptive interaction, emotion estimation

## Abstract

**Highlights:**

This study explores how robots can interact with people in a more engaging way. By combining real-time user engagement estimation with advanced language models, the system allows robots to respond consistently through both speech and body language. Tests show that this approach makes interactions feel more natural while improving user engagement and task success.

**What are the main findings?**

- Adapting the interaction based on user engagement significantly enhances user experience.
- The MiRo-E social HRI platform lends itself well to integrating verbal and nonverbal HRI.

**What are the implications of the main findings?**

- Enhancing perceived naturalness is an important goal in social human–robot interaction.
- Generative AI and multimodality offer a credible pathway to achieving this goal.

**Abstract:**

Adaptivity is a key component of social human–robot interaction (HRI) towards achieving more natural and human-like interactions. Current interactive systems tend to rely on preset and repetitive verbal communication and isolated nonverbal interactions, which results in unappealing engagement. This study proposes an integrated framework that combines a coordinated nonverbal interaction system based on real-time emotion expression with a fine-tuned large language model-based verbal communication system, resulting in more engaging and context-aware interaction. The design utilises the MiRo-E as the zoomorphic social interaction platform, with the aim of enhancing the consistency across verbal and nonverbal modalities and improving user engagement through adaptive and emotionally aligned responses. To evaluate the effectiveness of the approach, a user study was conducted with tasks designed to assess user engagement, task performance, and the perceived naturalness of interaction. Task performance metrics and subjective questionnaire responses indicate that the framework significantly enhances user experience, improving task completion rates, engagement, and perceived naturalness.

## 1. Introduction

From an early age, humans learn to pick up on subtle cues such as tone of voice, facial expressions, and verbal and nonverbal cues, and they adjust their own behaviour in response [1]. For instance, smiling back, mirroring posture, responding to verbal information, etc., are not calculated strategies but deeply ingrained, intuitive responses [1]. In the context of human–robot interaction (HRI), making the interaction *natural* is a key research goal [2,3]. Making robots interact in more human-like ways is seen to directly affect users’ interaction experience, sense of engagement, and willingness to interact [4]. Although social robots have widely appeared in daily life, current systems still have difficulty in offering engaging interactive experiences [4,5]. These systems often rely on fixed interaction behaviours, e.g., repetitive verbal communication, isolated and limited nonverbal cues, etc., regardless of the user’s state or the complexity of the task [2,4], limiting the appeal of the interactions.

Among the various modalities involved in HRI, verbal communication remains the most direct and expressive channel for conveying intent and building social rapport [2,6]. Nevertheless, nonverbal communication, including body language, touch, and spatial movement, also plays a vital role in interaction [7,8]. Henschel et al. [9] demonstrate that autonomously engaging in consistent and bidirectional communication helps achieve the so-called naturalness in interactive systems. In recent studies, emotional resonance has been identified as an important mechanism for resolving consistency issues [10,11,12]. Even so, an increase in interactive content or channels does not always lead to improved engagement [13]. Relatedly, “instructional scaffolding theory” in educational psychology [14] emphasises tailoring support to a learner’s ability and the complexity of tasks, which has shown promise in HRI [5]. However, little research explores the balance between minimal scaffolding support (open-domain dialogue) and highly structured support (task-oriented) in HRI.

Building on these concepts, this article proposes to enhance user engagement in HRI by ensuring consistency across verbal and nonverbal modalities with an integrated framework that combines real-time emotion expression with context-aware verbal communication. This integration addresses the bidirectionality and consistency requirements and aligns verbal and nonverbal communication while varying the interaction in recognition of the users’ level of engagement. Our approach is validated through a user study where the human interacts with a robot playing a story guessing task using a gamified approach. Given the entertaining nature of the user study, a zoomorphic social robot, the MiRo-E [15], is chosen for deploying the proposed framework, as it allows for more playful and multimodal interactions. Further, a multimodal audio–video-based system allows for estimating the user’s emotion in real-time. This article makes the following contributions:

(1) A framework for adaptive interaction based on user needs and task complexity.
(2) A dedicated emotion expression system for the MiRo-E using its various actuators and sound output systems.
(3) A component framework integrating fine-tuned large language models (LLMs) and engagement-aware communication functionalities.
(4) A systematic evaluation and analysis of the effectiveness of the adaptive framework with task-driven varying interaction levels in HRI scenarios.
(5) An audio–video-based real-time emotion estimation system using MiRo-E’s vision and audio system.

### 1.1. Related Work

#### 1.1.1. Adaptivity in Social HRI

Adaptivity has been a key research area in HRI in general, and previous studies have proposed a range of approaches. Aylett et al. [16] implemented an adaptive empathic robot tutor to support students using a rules-based adaptation behaviour, showing that adaptive behaviour can positively influence user engagement and experience. Tanevska et al. [17] introduced an adaptable *comfort* construct with the iCub robot, where the comfort increased when the robot perceived longer interaction from a human and decreased in its absence, with participants’ interactions varying noticeably as the robot varied its comfort level. Donnermann et al. [18] could not conclusively establish the superiority of adaptive interaction over a non-adaptive version in a higher education setting, showing that adaptive strategies need careful design. Andriella et al. [19] introduced the CARESSER adaptive framework implemented in a robot, combining therapist knowledge and patient interaction data (e.g., patients’ cognitive abilities) to provide tailored assistance, which demonstrated the potential for adaptive social robots in providing personalised and effective support. Bhat et al. [20] showed that an adaptive-learner strategy, which aligned the robot’s behaviour with human preferences, led to the highest levels of trust in a human–robot teaming setup. Overall, these studies report improvements in engagement, trust, and/or task performance. However, they also caution that not all adaptive strategies automatically lead to improved user experience or task outcome. Notably, they employ adaptivity using rule-based adaptation [16,17,18], inverse reinforcement learning [19,20], etc., applying their approaches in educational, therapeutic, or collaborative contexts. Relatively little attention has been given to gamified, narrative-based interactions.

#### 1.1.2. Multimodality in HRI

Researchers have increasingly focused on multimodality for nonverbal expression, including voice, image, text, eye movement, touch, etc. [21,22]. In terms of nonverbal cues, Urakami et al. [7] showed that nonverbal interaction is mainly visual (appearance, facial emotions) and auditory, where elements like pitch, tone, and language patterns significantly influence users’ perceptions of the interaction. The colour and type of a robot’s clothing and accessories can influence participants’ initial impressions even before the interaction begins [23], as do body movements [24], eye contact [25], and gestures [26,27]. Auditory nonverbal communication extends beyond the understanding of words to include interjections and pauses in speech, which have been shown to improve user attention during communication and interactions with robots [28]. Heredia et al. [29] pointed out that *emotion* can be the thread that binds together the multimodal outputs of social robots, i.e., a flexible emotion estimation and expression framework for better coordination. Peca et al. [30] chose to express emotions through the body swaying of the Keepon robot, which makes it easier for human users to communicate and share their thoughts. Researchers have also explored estimating and expressing emotions through the robot’s auditory systems [31]. Yamamoto et al. [32] successfully achieved human voice emotion estimation using pitch, speech speed, and intervals and enabled the robot to express emotions using synthetic voices. Nevertheless, significant gaps remain in the state of the art regarding the integration of emotion expression with LLM-driven communication for real-time interactive behaviours.

A related strategy is to make robots more aware of the user state during HRI, e.g., video, speech, and physiological (e.g., gaze, EEG, ECG, EMG, heart rate, etc.) and nonverbal signals [21,22,29]. The authors in [33], in one of the early instances of user state-based adaptivity, show eye gaze tracking for attention estimation and thus modulate the robot’s interaction. Bussolan et al. [34] show how multimodal fusion of physiological signals with facial videos and voice features is used to estimate the stress levels of operators in an industrial human–robot collaboration setting. Kothig et al. [35] use heart-rate variability to vary the interaction with an exercise coach-styled humanoid robot, while Shu et al. [36] provide a comprehensive review of physiological sensing for human emotion estimation, including a systematic review of datasets, features, and classifiers. More recent work by Garcia et al. [37] combines video, audio, and transcribed audio to text with pre-trained neural networks for emotion estimation tailored to specific users. This trend does establish the promise of multimodal sensor fusion for user state estimation for the robot’s interactive behaviour.

#### 1.1.3. LLMs for HRI

The recent evolution of AI models in advanced reasoning, semantic analysis, and contextual memory capabilities has enabled social robots to more flexibly understand user intent based on context and generate more natural conversations [38,39]. This can be leveraged for enhancing the adaptivity in social HRI—the focus of this article. Kang et al. [40] developed an LLM-driven system for social robots with human-like memory and affective capabilities. Their framework integrated retrieval-augmented generation to access chat history to improve response quality and leveraged the LLM to determine users’ emotions. Tang et al. [41] leveraged LLMs to integrate personality traits and memory to generate the robot’s personality and emotions, as well as adjust its behaviour based on past interactions. Spitale et al. [42] introduced an LLM-based robotic well-being coach that delivers coaching exercises by adapting to users’ multimodal behaviours. Hanschmann et al. [43] leveraged LLMs to implement the robot’s verbal and nonverbal communication, as well as enable real-time adaptation to consumers’ behaviour in a retail sales setting, demonstrating that it significantly increased consumers’ purchase intentions. Although demonstrating the potential of using LLMs in social robots, previous studies have mainly evaluated the effectiveness of LLM-powered robots in a specific field, and often lacked fine-tuning for adaptivity, or integration with other features, e.g., emotion expression, to simulate varying levels of interaction.

#### 1.1.4. Research with Zoomorphic Platforms: The MiRo-E

As noted in [44], different interaction tasks require robots with varying appearances. Machine-like robots are more suitable for tasks with low interactive requirements and high functionality, such as security, while zoomorphic robots, designed to resemble animals, are deemed more suitable as therapeutic companions and low-threat platforms for studying social bonding and learning, e.g., entertainment tasks, due to their playful and friendly appearance.

Zoomorphic platforms, e.g., PARO [45] and Huggable [46], have become important in social HRI research and real-world deployment. PARO [45] is a soft, seal-shaped robot, having sensors (tactile, visual, auditory, temperature, posture) and actuators (touch, voice, light, motion, eye blinks, and sounds), and it is mostly used in therapeutic and dementia care. AIBO, one of the earliest robot pet dogs designed by Sony [47], has been involved in social HRI research over a long term, exploring attachment, how people attribute personality to robotic pets, etc. It integrates perception, actuation, and modern AI algorithms for facial recognition, speech detection, emotion expression (sounds, motions, eyes), and navigation. PLEO [48], which is a commercial baby dinosaur toy robot, has also been used in HRI research and in longitudinal studies to examine play and changing engagement patterns between “toy” and “pet” roles. The Keepon robot [30], and its latest version, My Keepon, with its deliberately minimal, rounded form and simple motion/sound responsiveness, has been used in HRI research, facilitated by its simple, nonverbal interaction. Huggable [46] is a soft, sensor-rich companion robot, recently being explored in more complex HRI research, e.g., human–robot–human interactions, exploiting its soft body and dense sensing to allow touch-based affective social interactions.

In this article, the MiRo-E is chosen as the platform in this research for a narrative-style interactive task. The MiRo-E is a biomimetic, dog-like robot, similar to the AIBO, designed to mimic the expressiveness and behaviour of small mammals [15]. Its architecture combines a brain-inspired control system with multimodal sensing (vision, touch, auditory), enabling lifelike movements and emotional signalling [27]. Different from the other zoomorphic robots, the MiRo-E offers a good balance between expressive capability and open-sourced software architecture, which is important for controlled research implementations. The multimodality of this robot has led to some key research in social HRI. Barber et al. [10] expressed the MiRo-E robot’s emotions through sound, e.g., high-pitched and fast barking represents happiness, low-pitched barking represents anger, and a snoring sound that represents the MiRo-E falling asleep. They showed that the MiRo-E is likely to be more attractive for games and companionship tasks. Similarly, Ghafurian et al. [49] referred to behaviour patterns of multiple mammals, including dogs, rabbits, and mice, to design action combinations for the MiRo-E to express 11 different emotions, simulated by changing the MiRo-E robot’s eyelids, neck angle, head orientation, ear movement, tail shaking frequency, and LED lighting effects, supplemented by preset motion trajectories. Furthermore, studies have also explored how to use lighting effects and head orientation to indicate MiRo-E’s attention [50].

### 1.2. Scope of the Research

The article proposes an adaptive framework for a gamified HRI study and situates itself at the intersection of multimodality for nonverbal communication and context-driven LLMs as the core mechanism for direct verbal response generation. With the foundational assumption that more human-like interaction tends towards more natural interaction [2], this research uses emotion as the coordinating element to offer enhanced engagement. Emotional coordination helps sustain affective engagement beyond the novelty of social HRI, building trust owing to the naturalness of the robot’s behaviour [29]. Lack of emotional expression is shown to limit adaptation and reduce engagement [51].

It is important to note that generalisation in human-like interaction design tends to ignore the differences between individuals and tasks [52]. For example, educational robots provide unnecessarily detailed guidance when interacting with children who possess strong learning abilities [13]. Evidently, applying this design to an industrial setting would not be prudent, where more robot-like, focused interaction can be critical for productivity and efficiency [53]. To address this limitation, Schodde et al. [54] point out that the instructional scaffolding theory [14], where support is varied based on the current abilities of the learner, can be extended and integrated into the design of social robots. Their interactive system provides more detailed and verbose explanations to slower users, while lowering the verbosity for users showing a quicker grasp of ideas [54].

In addressing these key aspects, this article aims to provide valuable experience for the future design of social HRI with zoomorphic robots for more engaging interactions. The research closest to this approach was presented recently in [27]. That work explores the integration of LLMs with MiRo-E’s perception–action architecture to create more natural interactions. The authors showed that the MiRo-E can hold context-aware conversations grounded in real-time multimodal sensory input (vision, tactile, and speech), combining verbal input with information about the environment, user gestures, and tone. As will be seen later, in this article, in contrast to [27], a systematic user study is implemented, showing the effectiveness of the LLM-driven verbal response and emotion expression systems, demonstrating that the proposed multimodal adaptive framework significantly enhances social HRI with the MiRo-E. Further, an emotion estimation stack is also integrated, giving a real-time understanding of the user’s state.

## 2. Materials and Methods

The design of the integrated interactive system, covering the overall architecture, the adaptive verbal and nonverbal framework, MiRo-E’s emotion estimation and expression system during the task, and the user study setup, is presented here.

### 2.1. Overall Architecture

**Figure 1:**
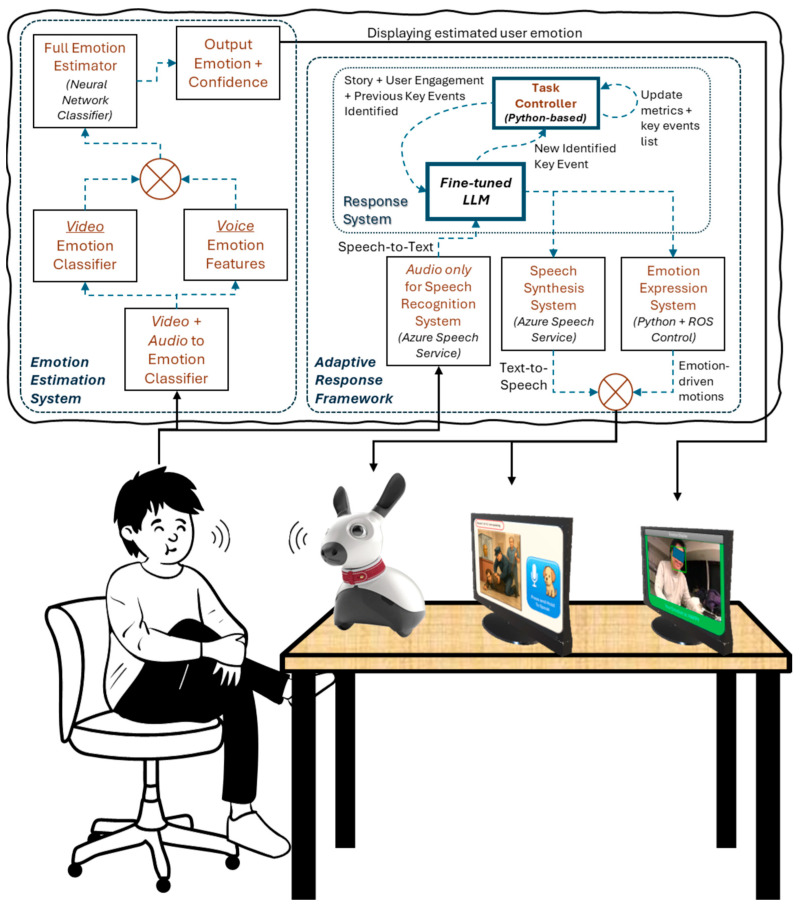
Overall architecture diagram.

Following the approach discussed in Section 1, the overall architecture consists of several components built around a core “response system”. The components include the adaptive framework based on speech synthesis and emotion expression systems and the central fine-tuned LLM-based response system. The emotion estimation system is based on real-time video and voice capture and feature estimation. As noted earlier, the MiRo-E platform, with its advanced sensing and actuation systems, is used as the perception and interaction system. Figure 1 illustrates the system architecture. The following subsections describe each system in detail.

#### 2.1.1. Speech Systems: Recognition and Synthesis

The verbal communication capabilities of the interactive system consist of three core stages: speech recognition, response generation, and speech synthesis. This design ensures real-time, bidirectional verbal interaction between the user and the robot. It also establishes a foundational framework for the subsequent integration of fine-tuned large-language models and emotion-aware communication functionalities.

The speech recognition system is the front-end subsystem that directly receives the user’s voice input through MiRo-E’s built-in microphones. To improve recognition accuracy, extra voice enhancement techniques are added, including noise reduction, echo cancellation, and automatic gain control, along with using only the data from left and right microphones. These enhancements ensure reliability and minimise background noise, improving the clarity of captured audio and reducing transcription errors in typical everyday conditions. The captured audio is written into an input audio file representing the user’s voice and processed by the Microsoft Azure Speech Service API [55], which was chosen for its robust performance and real-time transcription capability, converting the speech into text. This text serves as input for the response system.

The response system block consists of a large language model, conditionally fine-tuned on the textual input coming from the user, towards enhancing the naturalness of the interaction. The structure of this block is detailed separately.

The output of the response block is given to the speech synthesis system, where again, Microsoft Azure’s Text-to-Speech (TTS) service [55] was applied to convert the generated textual responses back into audible speech. After obtaining the generated audio, its sampling rate is manually adjusted to 8000 Hz to ensure speech clarity while reducing data size and facilitating real-time playback on the MiRo-E. The synthesised speech is then played back through MiRo-E’s built-in speaker, completing the verbal interaction loop. It is worth noting that the Azure TTS service used in this stage supports flexible adjustments of speech style, pitch, speaking rate, and volume. This capability provides a technical foundation for the subsequent implementation of the emotion expression part as well.

#### 2.1.2. Multimodal Emotion Expression System

A key factor in consistently integrating verbal and nonverbal communication is the ability to express emotions [7,27,49]. For the MiRo-E, it expresses its emotions through the cooperation of multiple modalities, including visual, auditory, and tactile feedback. In addition, the Microsoft Azure speech synthesis stage allows different styles, tones, and speaking speeds. The MiRo-E can combine changes in body posture, LED lights, and spatial movement with modulations in voice responses based on identified emotions.

**Figure 2:**
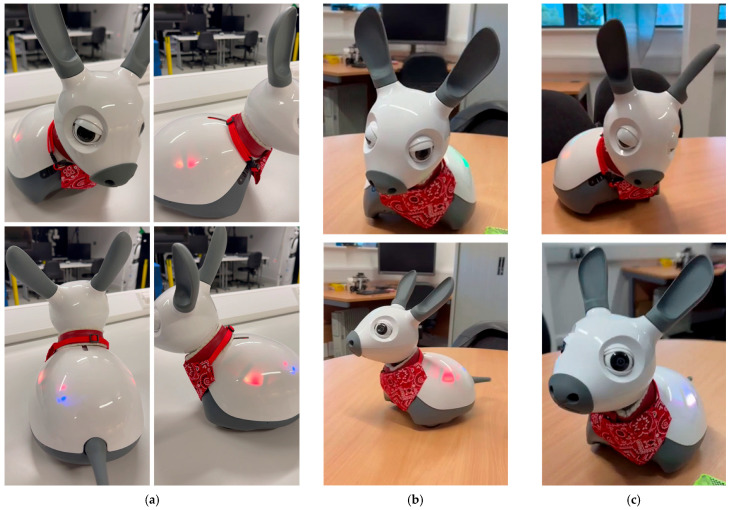
The MiRo-E showing the five emotions of happiness, calmness, excitement, sadness, and surprise. (**a**) Happiness. (**b**) Calmness (**top**), Excitement (**bottom**). (**c**) Sadness (**top**), Surprise (**bottom**).

As an example, Figure 2 shows snapshots of the MiRo-E in a happy state, making happy dog barks, wagging its tail quickly from side to side, quickly shaking its ears, spinning around, and replying with a more cheerful tone of voice. If the emotion of the MiRo-E is anger, the LED lights display a bright red colour combined with rapid flashing patterns. In terms of body language, the robot performs fast and full-range tail wagging, frequent head movements, and keeps its ears raised to simulate alertness and tension. In addition, MiRo-E’s spatial movement becomes more dynamic, moving rapidly in short bursts. Its auditory feedback is also adjusted, where the MiRo-E produces sharp and low-pitched dog barks, and its verbal responses adopt a faster speaking rate with an angry style. Taking inspiration from the work done by Ghafurian et al. [49], 11 different emotional expression models were implemented, all of which made clear distinctions in visual effects, body language, and auditory expressions, showing *happiness*, *excitement*, *sadness*, *fear*, *disgust*, *surprise*, *calmness*, *boredom*, *annoyance*, *anger*, and *tiredness*. Table 1 lists the poses and motions that the MiRo-E does when executing these expressions. Figure 2 also shows snapshots of the MiRo-E exhibiting the distinct behaviours when expressing the emotions of happiness, fear, tiredness, and anger. The implementation of the emotions on the MiRo-E is done through a Python 3 based middleware control layer using Robot Operating System (ROS) (version ROS 1 Noetic), which allows MiRo-E’s physical movements to be triggered directly from the overall framework.

Tactile sensation is also incorporated into the design, where human users will get different responses when touching MiRo-E’s back in different states. For example, when in a sad state, touching (or caressing) the back of the MiRo-E will soothe its emotions, causing it to reopen its half-closed eyelids and raise its head again.

#### 2.1.3. Response System

As seen in Figure 1, the outputs of the speech recognition systems are fed to the central response system. Taking advantage of recent LLMs allowing context-aware conversations, the response system employs OpenAI’s gpt-4o-mini model. The gpt-4o-mini model was chosen primarily as it strikes a good balance between computational cost and offering strong language reasoning and generation capabilities compared to other models [56]. Another reason was its ready availability in the research group. The output of the response system drives the verbal and nonverbal components of MiRo-E’s interaction with the user. The emotion expression system processes the output to detect keywords (e.g., “Yes” or “No”) to execute the appropriate emotion model. Together, the speech output and emotional movements form MiRo-E’s integrated multimodal response mechanism, seeking to enhance the engagement of the interaction and providing users with a richer and more human-like experience.

It is to be noted that the gpt-4o-mini model needs to be fine-tuned to implement the adaptivity that is a central part of the proposed adaptive framework. As LLM fine-tuning itself is task- and context-dependent, the following sections first describe the context, i.e., the gamified interaction setup, with the details of the LLM processing following in Section 2.2 and Section 2.3.1.

#### 2.1.4. Emotion Estimation System

**Figure 3:**
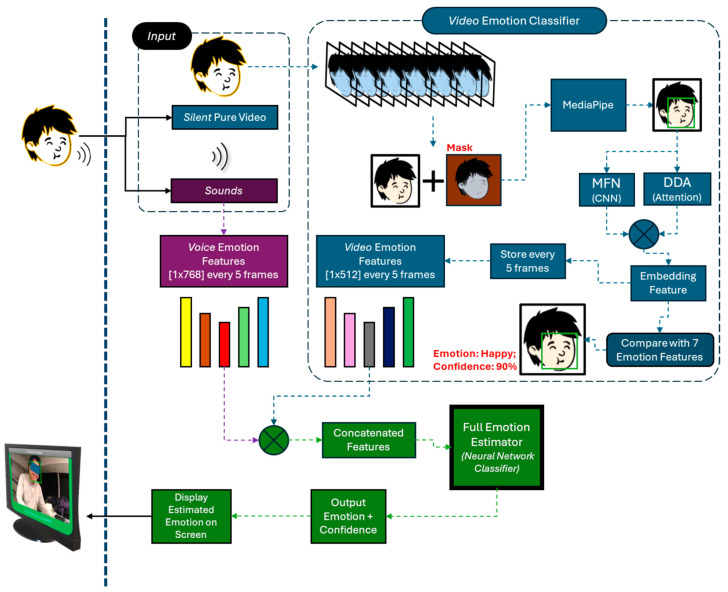
Multimodal emotion estimation system.

As noted earlier, emotion (estimation and expression) forms a key component of the proposed adaptive framework for verbal and nonverbal interaction. A multimodal model was developed to estimate user emotions in real-time by simultaneously processing audio and visual input. The system architecture is shown in Figure 3, and the implementation details are described below.

Keltner and Cordaro [57] suggest that emotions are complex behavioural patterns that involve multiple modalities: facial muscle movements, vocal cues and tonality, etc. For this system, the audio–video input from the MiRo-E observing the user is initially divided into two distinct streams and processed separately to extract distinct emotion features, which are then combined through feature splicing to determine the overall emotion class. To balance estimation accuracy and real-time performance, the system processes data every 5 frames, ensuring that the emotion estimation results are updated efficiently without introducing noticeable delays.

For visual emotion estimation, the video input is first processed using MediaPipe [58] to detect and crop facial regions, ensuring that only relevant facial expressions are analysed. The cropped facial images are then passed through a convolutional neural network (CNN)-based feature extractor, where convolutional layers capture local features related to facial expressions. However, emotional expressions frequently involve coordinated changes across multiple separated facial features. For instance, happiness is often expressed through the raising of the corners of the mouth and the formation of wrinkles around the eyes. To address this challenge, a coordinate attention mechanism is integrated into the CNN-based visual feature extractor. This mechanism enhances the model’s capacity to capture long-range dependencies by embedding spatial coordinate information directly into the feature maps [59]. Unlike traditional channel attention mechanisms, which primarily focus on global feature re-weighting, coordinate attention decomposes attention into two separate directions: horizontal and vertical. Through this enhancement, the visual emotion estimation part generates more discriminative visual features by capturing the holistic facial expressions associated with different emotional states. The attention-integrated CNN is implemented through the pre-trained DDAMFN model [60], which integrates the (i) Mixed Feature Network (MFN) lightweight CNN backbone specifically tailored for face verification tasks and (ii) the dual-direct attention head to construct attention maps based on extracted features. The pre-trained model shows a 67% accuracy on the AffectNet-7 dataset [61]. Finally, to avoid scale differences between visual and audio emotion features, the extracted visual features are standardised and output as 1 × 512 vectors.

For the audio part, the system adopts the pre-trained Emotion2Vec model [62], which provides a rich representation of emotional features from speech. As noted in [62], the model outperforms most baseline models with a weighted accuracy of around 85% on mainstream English datasets. The pre-trained original model is used as is, with only the output layer removed to obtain the high-dimensional intermediate features, which would be suitable for downstream fusion with the separately extracted visual features. These audio emotion features are standardised to 1 × 768 feature vectors to ensure consistency across scales and stability of the subsequent classification model.

The standardised audio 1 × 768 and visual 1 × 512 features are concatenated to form a unified 1 × 1280 general multimodal feature vector. This fused emotion feature is input to a customised deep neural network (DNN) classifier. This is a lightweight DNN that maps the 1280 input features through 3 fully connected hidden layers (512 → 256 → 128) with ReLU activations. Batch normalisation is applied after the first 2 layers, and a small dropout (*p* = 0.05) is applied before the final layer. The last linear layer outputs both the predicted emotion category and a corresponding confidence score. The validation of the model is discussed in Section 2.3.2.

### 2.2. Gamified Interaction Setup

As discussed earlier, Schodde et al. [54] demonstrate that varying levels of interaction based on the user’s requirements for interaction allow for more natural engagement with the user. Drawing on the research of [16,19,27,40,54], the interaction in this research focuses on testing the proposed adaptive framework against the baseline that does not incorporate adaptivity.

**Figure 4:**
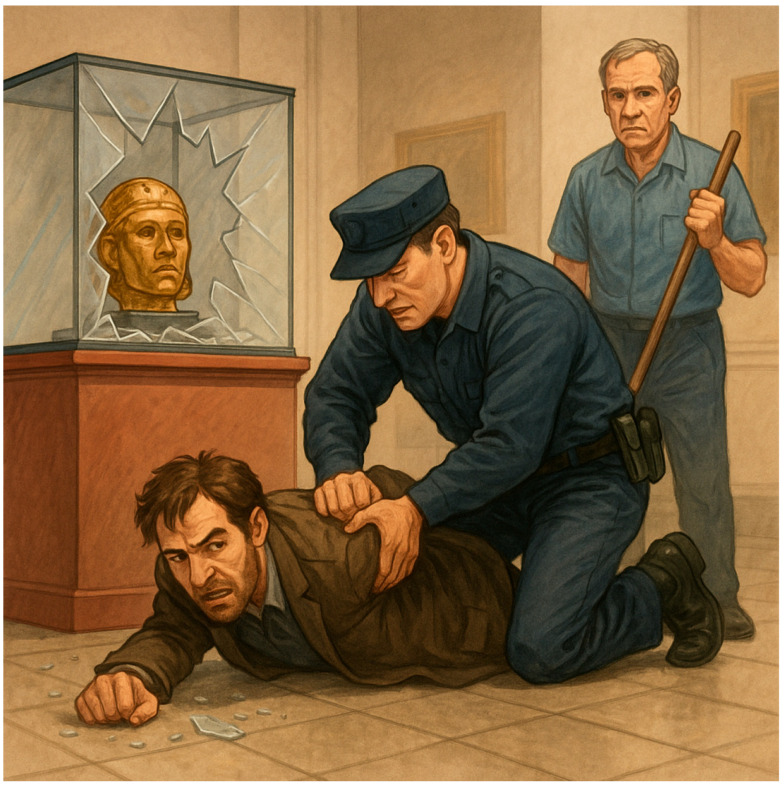
Example image of the story guessing task: Museum Thief and Janitor.

A gamified, interactive, story guessing task is deployed, involving visual reasoning, where the user interacts with the MiRo-E to determine the key plot points and narrative outline of the story behind the presented image. This task is inspired by a well-known puzzle, usually referred to as a *situational puzzle* or lateral thinking puzzle, in which players are presented with scenarios containing limited information and must ask targeted questions and make logical inferences to uncover the underlying story or solution. This requires a sophisticated level of cognitive processing, blending creativity, reasoning, and inference skills [63]. By examining how the MiRo-E with adaptive (or non-adaptive) behaviour can support users in solving such cognitively demanding tasks, insights can be gained into the extent to which the proposed framework enhances user performance and engagement. The story guessing task consists of a full narrative story, an image that conveys the ending of the story, and three key events of the story. Figure 4 shows an example of one of the stories. During the task, users are required to guess the three key events of the story based on what they see in the image. Users engage by asking yes/no questions to the MiRo-E, which responds by offering hints depending on the question. When a key event is correctly identified, the MiRo-E will acknowledge it, and once all three events are discovered, it will prompt the user to guess the full story by linking the key events together. The task is limited to a maximum of 12 rounds; a successful full story guess indicates successful completion of the task. On the other hand, failure to identify the full story within this limit is considered an unsuccessful attempt.

The two levels of interaction were designed as:

1. *Adaptive* mode: In adaptive mode, the MiRo-E’s generated response behaviours vary based on two main factors: (i) the user’s task performance, measured using the round number and number of key events successfully identified by the user, and (ii) the user’s engagement level, which is measured by the user’s response time. This is accompanied by the appropriate emotional expression output as well. The conversation chain is retained by the system as contextual memory to enhance the coherence and continuity of responses.
2. *Non-adaptive* mode: In non-adaptive mode, the response behaviours are consistent and static, deliberately limited in the amount of information shared to ensure brevity. Instead of offering direct hints or leading questions, the responses adopt evaluative statements, such as true or false. Also, the system refrains from providing overly direct or intuitive responses to the user.

For instance, if the current round number is 8 and the user asks “Did the janitor catch the thief?”, in the example story seen in Figure 4, the adaptive mode responds with: “No. Although the janitor played an essential role in alerting security, he did not physically catch the thief. He pressed a hidden alarm that was crucial in summoning help just in time!” In contrast, the non-adaptive mode replies with: “No, he didn’t catch the thief himself, but he did help prevent the theft by alerting others.” Both response modes are accompanied by selected emotional expressions, taken from Table 1, to allow a full interaction to take place. Section 2.4 describes this in more detail.

#### 2.2.1. LLM-Powered Text Corpora for MiRo-E Response Generation

To implement the adaptivity in the framework, the LLM is integrated with a traditional rule-based adaptation [64], allowing for the generation of different response behaviours based on real-time measured user performance and engagement levels while managing potential ambiguity in user inputs. For this integration, a response rule is provided to the LLM as a *system prompt*, which specifies the expected response behaviour from the LLM based on the users’ metrics. The response behaviour should adjust based on two main factors: the user’s task performance, measured using the round number and number of key events successfully identified by the user, and the user’s engagement level, which is measured by the user’s response time. The engagement level is classified as low if the user’s response time, defined as the interval between the end of the previous round and the user’s input in the current round, is longer than a threshold, which is defined as 10 s for this article.

**Figure 5:**
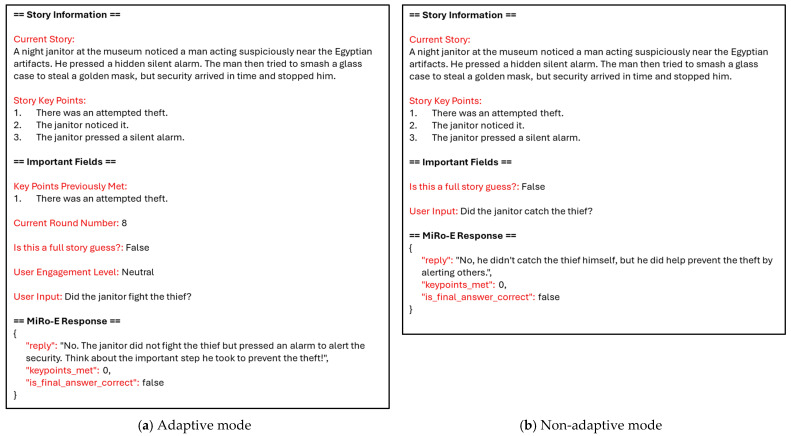
Part of the constructed user prompt for each condition.

In addition to this preset system prompt, which is provided to the LLM only once, a preformatted *user prompt* is generated for each round to guide the model’s response. This prompt includes the following information: the full story used in the current task, the key events of the story, the current round number, the key points previously identified by the user, whether the current round is a full story guess, the user’s engagement level, and the user’s input. For instance, the full story for the image in Figure 4 is “A night janitor at the museum noticed a man acting suspiciously near the Egyptian artifacts. He pressed a hidden silent alarm. The man then tried to smash a glass case to steal a golden mask, but security arrived in time and stopped him.” The key events are “There was an attempted theft.”, “The janitor noticed it.”, and “The janitor pressed a silent alarm.” When the user asks questions such as “Did someone try to steal something from the museum?”, it will be considered as meeting the first key event. Once all key events are met, the user can try to guess the full story by linking the key events. Inputs such as “A man is trying to steal something from the museum, the janitor noticed it and pressed a silent alarm” will be considered correct; meanwhile, inputs such as “A man is trying to steal something from the museum, the janitor noticed it and caught him” will be considered incorrect. Figure 5 illustrates examples of such user prompts to be sent to the LLM for the adaptive and non-adaptive modes. The story information is included in the user prompt message itself, which is altered based on the story being considered. Furthermore, instead of feeding the entire chat history to the LLM, the system provides only the key points previously identified by the user. This enables the model to generate targeted hints towards undiscovered key points while minimising memory requirements. Examples of the combined system + user prompt for both modes are included in the Appendix A.

The adaptive mode user prompt accounts for information, including the number of rounds completed, key points identified, and the user engagement level (neutral or low), which are used to generate hints to accompany the “Yes/No” responses of the model. Out of the 12 rounds permitted for the interaction, in rounds 1 to 3, the model only provides hints in case the user engagement is low. From rounds 4 to 7, the model begins to provide stronger hints if not enough key events have been identified. From round 8 onward, the model always generates strong hints regardless of how many key events have been guessed previously. Across all rounds, encouragement is incorporated into responses if the user engagement is low.

On the other hand, the non-adaptive mode user prompt only provides consistent hints and does not account for the factors considered for the adaptive mode. For both models, if the current round is a full story guess, the model will evaluate whether the full story provided by the user is correct. If the answer is correct, it will be acknowledged, and the task will end; otherwise, the user is prompted to try again.

#### 2.2.2. Response System—Integrated Task Controller

The dedicated task controller works in conjunction with the LLM block, as seen in Figure 1. In the implementation, the combined user input, story information, task metrics (e.g., round number and engagement level), and a record of previously identified key events are input to the LLM block as a formatted user prompt. The key events are stored in a Python list, which is dynamically updated as the task progresses. The JSON-formatted response contains the textual response and information on the key event and full story guessing, which are kept updated by the Python-based task controller. This ensures that user progress is maintained across rounds and that the system can generate contextually relevant responses. In parallel, the textual response is sent to the speech synthesis system for speech generation, while keyword detection triggers execution of the corresponding emotion model within the emotion expression system. The Boolean field indicating a successful full story guess determines whether the task should end. All logic for state management, task flow, and inter-component coordination is implemented in Python, which serves as the memory backbone and central controller of the system.

### 2.3. System Validation

This section discusses the validation of the models used in the response system and the emotion estimation system.

#### 2.3.1. Rules-Based Response LLM Fine-Tuning

The fine-tuning process involved designing structured system prompts (examples in the Appendix A) that define the expected response behaviour of each model. The system prompts were first tested in the OpenAI Playground to evaluate their effectiveness and to generate training examples for fine-tuning the gpt-4o-mini model. This process involved providing manually crafted user input messages to the model, observing its responses, and modifying the responses when necessary. The adjusted examples were then considered as training examples, formatted in a structured JSONL file, one for each mode, specifying the system prompt, user prompt, and MiRo-E message for each instance. The file for the adaptive mode consisted of 29 training examples: 11 for low engagement and 18 for neutral engagement. The non-adaptive mode file consisted of 18 examples. The JSONL files served as the dataset to fine-tune the base model.

**Figure 6:**
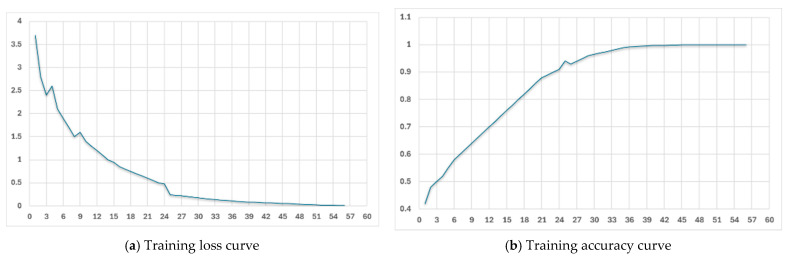
Training loss and accuracy curves for the adaptive mode corpora.

During the training process, the hyperparameters used for the fine-tuning process were: Epochs = 3, Batch Size = 1, Learning Rate Multiplier = 1.8, and Seed = 150,672,096. Figure 6 shows the training loss and accuracy curves for the adaptive mode fine-tuning (similar curves were obtained for the non-adaptive mode). The training loss decreases rapidly and then smoothly over the steps, and the training accuracy also reaches over 95%, illustrating that the model successfully captures the target’s conversation mode. After fine-tuning the same model on the two corpora separately, two different models were obtained: gpt-adaptive and gpt-non-adaptive.

#### 2.3.2. Emotion Estimation Model Training

**Figure 7:**
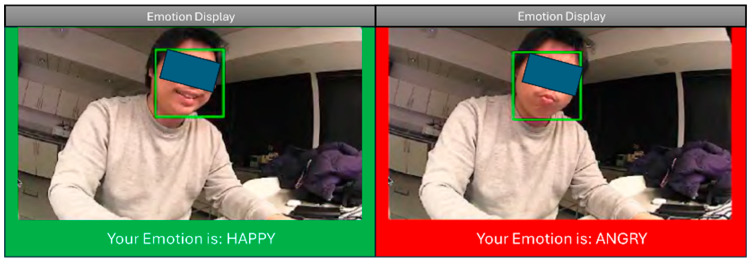
UI interface showing emotions based on MiRo-E camera video stream.

The basic structure of the model established in Section 2.1.4 is trained and tested to validate the emotion estimation approach. The Ryerson Audio-Visual Database of Emotional Speech and Song (RAVDESS) [65] was selected for the training and testing. The RAVDESS is a validated multimodal emotional speech and song database [65]. It contains 7356 files of combined facial and voice recordings of 24 professional actors expressing 7 different emotions in English—happiness, sadness, anger, fear, surprise, disgust, and neutral. The dataset was split 80–20% for training and testing. After the feature extraction process, the set of emotion features for the seven distinct emotion labels was obtained. The model achieved an F1-score of 0.89 on the testing dataset and an average of 89% accuracy across all seven emotion categories without any significant bias toward any emotion category. Figure 7 shows a simple display of the detected emotions using different colours in real-time.

### 2.4. User Interface Design

**Figure 8:**
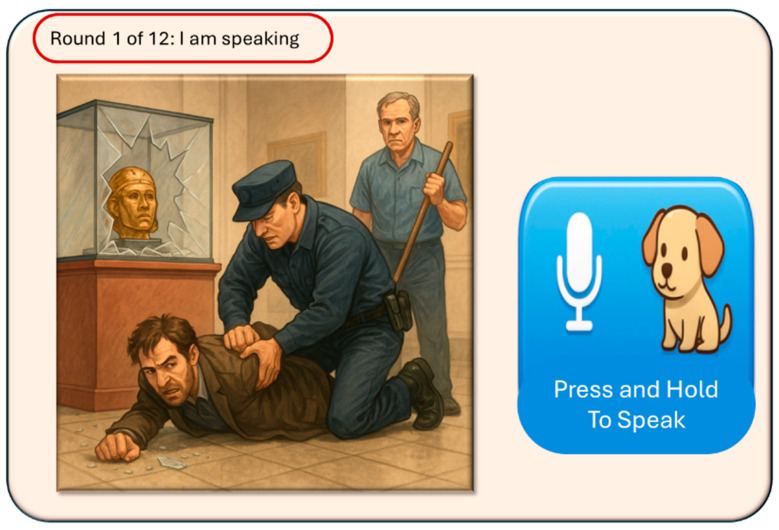
User interface designed for the story guessing task.

Following the validation of the response system framework, the user interface for the story guessing task was designed to be simple and effective, presenting all necessary information on a single window. Figure 8 illustrates an example of the interface used in the task. The interface consists of three main components: a status bar on top, the story image, and a press-and-hold button for speech input. The status bar dynamically updates the round number and MiRo-E’s current status in real time. For instance, when the MiRo-E is speaking, it displays “I am speaking”; when the user is recording input, it displays “I am listening”; and when the system is processing input, it displays “I am thinking”. The story image is presented in a sufficiently large format to allow users to clearly observe and interpret its details. Finally, the press-and-hold button is designed to enable convenient speech input, allowing users to record their voice seamlessly during the task.

In addition to visual cues, audio cues were incorporated into the interface to enhance user-friendliness. At the beginning of the task, the MiRo-E delivers a welcoming speech that provides clear instructions and ensures users have sufficient information about the task. The speech is as follows: “Welcome to the Story Guessing Task! The objective of this study is to guess the story based on the things going on in the image you see on your screen. Your goal is to uncover the three key events of the hidden story. You can ask me yes or no questions to gather clues and try to guess each key event as you go. Whenever you correctly identify a key event, I’ll let you know. Be sure to pay attention to any hints I give, they’ll help you along the way! Once you’ve discovered all three key events, I’ll ask you to piece them together into a full story. The maximum number of rounds is 12. Let’s get started!”

During the task, the MiRo-E provides additional verbal feedback. For example, when processing user input, it responds with “Thanks for the input! Let me think about that!” Upon task completion, the MiRo-E adapts its closing speech to the outcome. If the user successfully completes the task, it congratulates them and reads the full story aloud; however, if the user fails, it will offer reassurance by saying “You did your best!” before reading the full story.

Furthermore, out of the designed emotional expressions in Table 1, five were used for the user study: happiness, excitement, surprise, sadness, and calmness.

- Excitement, including the playful winking, corresponds to every time there is a correct response, which occurs when the user successfully guesses a part of the story, triggered when the system’s response contains the keyword “Yes” or when a key event is identified.
- If the participant completes the task within four rounds or fewer, the MiRo-E will trigger the surprise emotional response, where the MiRo-E delivers an excited vocal response using a surprised tone, saying “Wow, you got the answer already? That’s amazing!”
- The sadness emotion corresponds to an incorrect response, triggered when the user fails to identify a part of the story during a given round. This occurs when the system’s response contains the keyword “No”, when the user incorrectly guesses the full story, or when the task is ultimately failed.
- Happiness is triggered when the user successfully guesses the full story, conveying the user’s success in completing the task.
- Calmness emotion is triggered when the user begins to speak (by pressing the button on the interface), and this emotion is maintained when the user’s input is completed and the robot is thinking.

On top of this, blinking orange lights are added to all emotions when the MiRo-E is speaking and until the end of its speech, when the user’s engagement level is detected to be low. The colour orange is perceived as warm and energetic, which may help convey encouragement.

### 2.5. User Study Design

To evaluate the effectiveness of the proposed adaptive approach, a user study was conducted with 10 adult participants, all from the university’s MSc international student cohort. Using a within-subject study design, each participant was asked to complete the task twice, once in adaptive mode and once in non-adaptive mode, in sequential order. Their preferences, together with performance and perceived user experience, were evaluated to compare the two modes.

Before the study began, participants were asked for their informed consent and to complete a pre-study questionnaire, which briefly described the purpose of the study, provided instructions for the task, and evaluated whether participants understood the study’s objectives and procedures. Based on the responses, none of the participants had prior experience of interacting with a robot or had participated in any HRI study. Afterwards, participants were guided through detailed instructions about the task, including how to interact with the task interface and what kind of input should be given. Prior to starting the actual study, they were given the opportunity to complete a trial task to familiarise themselves with the task and interface. This trial was designed to minimise the effect of task non-familiarity on the study results. Participants were allowed to repeat the trial task as many times as needed until they felt comfortable proceeding to the main study.

Three stories were designed for the user study: one for the trial task, one for the adaptive mode, and one for the non-adaptive mode. This allocation ensured that all participants were exposed to the same story in the same condition, thereby minimising potential variability caused by differences in story content. Assigning a dedicated story for the trial task further ensured that participants’ practice attempts did not overlap with the actual evaluation tasks, preventing familiarity with a particular story from influencing their performance during the main study. Five participants (P1, P2, P5, P6, P7) interacted with the adaptive mode first (AF group), while the other five participants (P3, P4, P8, P9, P10) began with the non-adaptive mode (NAF group). The details of the model itself were not conveyed to the participant—they were masked as “first mode” and “second mode” for the purposes of the user study. The three storylines and key events are listed in Table 2.

#### 2.5.1. Ethical Statement

The study was conducted in accordance with the Declaration of Helsinki and was approved by the Ethics Committee of the School of Computer Science at the University of Nottingham, UK (ID CS-T-2025-SOP1.1-ND-PY-20386366), with a date of approval of 23 June 2025.

#### 2.5.2. Objective Evaluation Metrics

The objective evaluation was based on participants’ task performance by two indicators:

- Task completion rate, which represents the proportion of participants who successfully guessed the full story within the allowed number of interaction rounds.

$$TCR\;\left(\%\right)=\frac{Number\;of\;successful\;participants}{Total\;number\;of\;participants}\times 100$$

- Average number of rounds for task completion, which refers to the average number of rounds to successfully guess the full story, within the maximum number of rounds.

$$ANR=\frac{Sum\;of\;Number\;of\;Rounds\;for\;Task\;Completion}{Total\;number\;of\;successful\;participants}$$

- Task completion time (seconds), which represents the time taken by participants to either successfully complete the task or fail the task at the maximum 12-round threshold.

#### 2.5.3. Subjective Evaluation Metrics

Following the completion of both conditions, participants were asked to fill out a post-study questionnaire. Inspired by the System Usability Scale (SUS) [66], the questionnaire consists of statements to be rated on the standard as well as a double-ended 5-point Likert scale, comparing users’ preference for the “first mode” vs. “second mode”, with a mixed positive and negative phrasing to reduce response bias. The questionnaire, shown in Table 3, was divided into 3 categories, having questions assessing the users’ perceived experience during the story guessing task, the quality of MiRo-E’s responses, and the effectiveness of MiRo-E’s emotional expressions. The responses from the first two categories of the questionnaire serve as the main subjective measures for evaluating the effectiveness of the adaptive mode, with participants’ preference for the “first mode” or “second mode” mapped to the corresponding model over the 5-point scale as “Strongly Adaptive (1),” “Weakly Adaptive (2),” “Neutral (3),” “Weakly Non-adaptive (4),” and “Strongly Non-adaptive (5)”. Meanwhile, the third category is primarily used to assess MiRo-E’s emotion expressions, to provide insights that may inform the future design of emotional behaviours in social robots. It is important to note that at this stage, the estimated emotion (from Section 2.1.4) is not included in the subjective evaluation, and, therefore, is not evaluated for its usability in this study.

Given the small sample size, the data distribution from the questionnaire did not satisfy the assumptions of analysis of variance. Therefore, to establish the significance of the results, two non-parametric tests are used:

1. The Wilcoxon rank-sum test [67] is used to test the two hypotheses:
  - H1: “AF and NAF groups both consistently prefer the adaptive mode”;
  - H2: “AF and NAF groups both consistently rate the MiRo-E emotion expressions positively.”

The medians of the assigned scores for both groups were used for this comparison.

2. The Pearson chi-squared test for goodness of fit [68] was used to test the frequency distribution of the assigned scores, which accommodates a comparison of 2 sets within the same sample condition [69]. Here, the sets are the frequencies of favourable and non-favourable scores assigned to the questions. For instance, in C1, a score of “1” for questions Q1-Q2-Q4 would be *favourable* for a strong positive evaluation of the adaptive mode. Any other score is considered *non-favourable*. Similarly, in C3, a score of “1” for scales Q1-Q2-Q4 and a score of “5” for Q3 would be favourable, implying a positive evaluation for MiRo-E’s emotional expression.

## 3. Results

The results of the user study, including objective task performance metrics and subjective measures, are presented here.

### 3.1. Objective Measures

**Figure 9:**
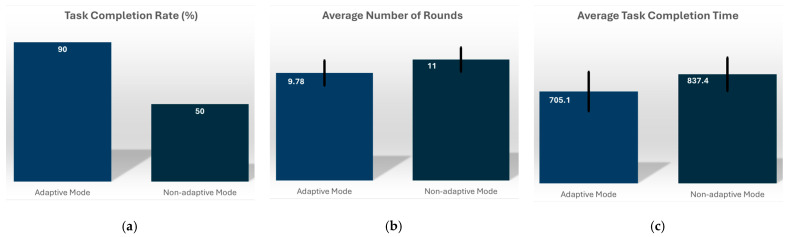
The objective measures are captured in this figure: (**a**) task completion rate (%); (**b**) average number of rounds for task completion; (**c**) average task completion time (seconds).

Figure 9 shows that the task completion rate for the adaptive mode was significantly higher, 90%, compared to 50% for the non-adaptive mode. Meanwhile, the average number of rounds required for task completion was 9.78 rounds for the adaptive mode and 11 rounds for the non-adaptive mode. A closer breakdown of the results shows that:

- Four participants completed the task using the adaptive mode but failed to complete it with the non-adaptive mode;
- Four participants completed the task in both conditions but required fewer rounds with the adaptive mode;
- One participant failed to complete the task under either condition;
- One participant completed the task in the same number of rounds in both conditions.

The average completion time shows the adaptive mode taking a much shorter time than the non-adaptive mode by about 132 s. Although this is a large difference, it is not statistically significant using the paired Student’s t-test at the 5% level (*p*-value = 0.0585).

### 3.2. Subjective Measures

**Figure 10:**
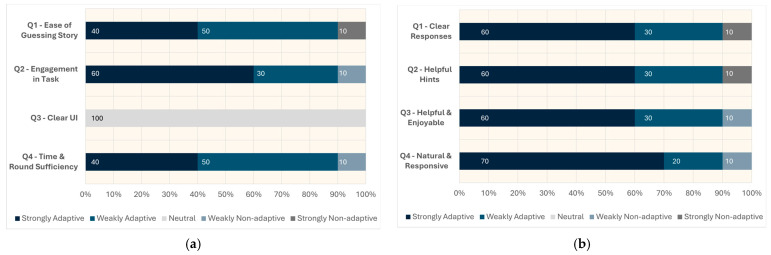
The subjective measures are captured in this figure: (**a**) C1-Q1 to Q4; (**b**) C2-Q1 to Q4.

**Figure 11:**
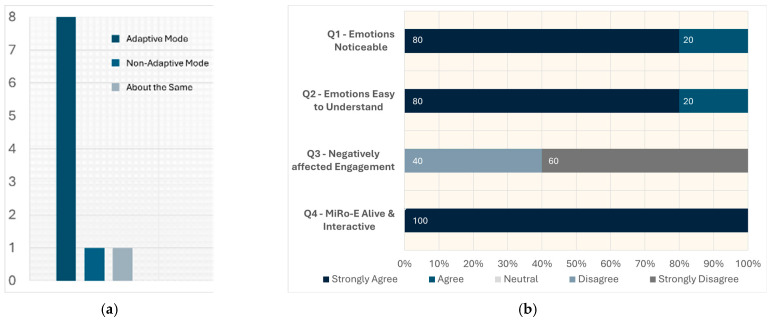
(**a**) Preference between adaptive and non-adaptive modes. (**b**) Subjective assessment of MiRo-E’s emotional expression.

Responses from the first and second categories of the post-study questionnaire, with the double-ended Likert scale questions, are shown in Figure 10, while participants’ overall preferences between the two models are summarised in Figure 11. The qualitative feedback from the open-ended questions is discussed subsequently.

The Figure 10a results clearly lean towards the adaptive mode for Q1, Q2, and Q4, with only 10% of responses favouring the non-adaptive mode. Figure 10b presents findings similar to those from C1, showing a clear preference toward the adaptive mode, with leanings exceeding 50% across all questions. Meanwhile, almost all participants selected the neutral option for Q3, as the user interface was identical across both modes.

Both categories asked open-ended questions. C1 asked about any improvements to the story-guessing task itself. A few participants commented that they wish to have more chances to guess the story. One participant noted that both the response time and the length of responses were too long, commenting “Sometimes the robot thinks too long.” C2 asked about participants’ perceived difference between the two modes, with most participants noting that the adaptive mode provided more helpful and clearer hints, whereas the non-adaptive mode tended to repeat similar hints. Some participants also observed that the adaptive mode gave more encouragement during the task, while others highlighted that the adaptive mode’s responses were noticeably longer compared to the non-adaptive mode. The descriptive feedback is noted in Table 4.

Participants were then asked to indicate their overall preference between the two modes and to provide their reasons. As shown in Figure 11a, the majority of the participants (*n* = 8) preferred the adaptive mode, while one participant preferred the non-adaptive mode and another reported no clear preference.

#### 3.2.1. Emotional Expression of the MiRo-E

From the questionnaire, we also collected feedback regarding MiRo-E’s emotional expression, as described in Section 2.5.3. As seen in Figure 11b, all participants reported that the emotions were noticeable, clear, and easy to understand. All participants disagreed, six of them strongly so, that the emotional expressions of the MiRo-E negatively influenced their engagement. A total of 100% of the participants stated that the MiRo-E felt more alive and interactive due to its emotional expression.

#### 3.2.2. Statistical Results

Table 5 captures the results for the Wilcoxon rank-sum test. The “Score” column captures the mean and median scores assigned by the subjects for the corresponding statements. The “Rank Sum Value” and “*p*-value” columns show the test statistics.

Table 6 shows the results of the Pearson chi-squared test. The “Frequency of Score” column captures the number of subjects who assigned the favourable and non-favourable scores for the corresponding statements. For the chi-squared test, the expected frequency is 2 for the favourable score (10 subjects over a 5-point scale) and 8 for the non-favourable scores. The “$\chi^{2}$(1) Value” and “*p*-value” columns show the chi-squared statistics.

## 4. Discussion

Our findings point to the advantages of the proposed adaptive framework. The objective and subjective measures indicate robust differences between participant responses, showing a clear preference for the adaptive mode compared to the non-adaptive one.

### 4.1. Objective Measures

The adaptive mode achieved a higher completion rate compared to the non-adaptive mode and generally required fewer rounds to complete the task, suggesting that the adaptive framework was more effective in assisting participants in successfully completing the task and facilitated more efficient task progression compared to the non-adaptive mode. The time taken to complete the task was shorter when using the adaptive mode compared to the non-adaptive mode, although this was not statistically significant. This was also the case where a participant completed both tasks in the same number of rounds. This observation provides supplementary evidence that the adaptive framework supported more efficient task progression.

### 4.2. Subjective Measures

In category C1, a majority of the participants responded that the adaptive mode helped make story guessing easier, increased their engagement in the task, and it provided a stronger sense of assurance that the number of rounds was sufficient. From the users’ perspective, this response indicates that the adaptive mode was more effective in supporting performance in a complex task. Users gave neutral feedback for the interface design in Q3, which shows that it did not affect user engagement in the task, and MiRo-E’s behaviour perhaps played the major role in that. In C2, participants seemed to perceive the hints provided by the adaptive mode as more helpful, and their responses were seen as more enjoyable, natural, and responsive. For the overall preference, most participants favoured the adaptive mode because it helped them complete the task more effectively and offered helpful hints, while others preferred it because it felt more natural to them.

Table 5 helps establish the two hypotheses proposed. For categories C1 and C2, the mean and median values for both the AF and NAF groups being close to 1 (except C1-Q3) show that the adaptive mode is favoured over the non-adaptive one. Importantly, as all the *p*-values indicate, there is no statistically significant difference between the scores assigned by the AF and NAF groups, which proves hypothesis H1 that both groups consistently prefer the adaptive mode. Likewise, for C3, the *p*-values confirm that the scores assigned by the AF and NAF groups are statistically indistinct, which proves hypothesis H2 that both groups consistently rate the MiRo-E emotion expressions positively. Together, H1 and H2 prove that regardless of the user beginning the interaction in the adaptive mode or the non-adaptive mode, they show a significant preference for the adaptive mode and strongly acknowledge the positive influence of MiRo-E’s emotions on their engagement.

In Table 6, for C1 and C2, all scales favour the adaptive mode, as confirmed by the mean values being closer to 1 (Strongly Adaptive). Other than scales C1-Q1 and C1-Q4, the Pearson chi-squared test shows all the scales strongly favouring the adaptive mode in a statistically significant way. In C3, based on the statistical significance of the favourable scores, participants were unequivocal in their appreciation of the emotional expression and interactivity provided by the MiRo-E, positively supporting the users’ engagement. Collectively, these findings demonstrate that the adaptive mode did have a positive influence on the user engagement, which directly addresses one of the core research motivations of this study.

The responses to the open-ended questions were indeed informative. In particular, some of the key phrases from the unstructured feedback indicated that the adaptive mode felt “more encouraging,” “more engaging,” and “more natural” for the users, thereby improving the overall user experience. However, some issues were also identified that negatively affected this aspect. For instance, some participants perceived the responses in some cases to be quite lengthy for the adaptive mode—it is verbose by design. Nevertheless, the results demonstrate that users did not find it to be “wasting” their time. The study also highlights opportunities to further refine the adaptive mode towards improving the HRI experience. For the emotion expression, most participants particularly appreciated the “happiness” emotion displayed when they successfully guessed the full story. Others highlighted the nodding and head shaking behaviours as enjoyable, while a smaller number mentioned liking the wink, tail movements, and light blinking. This is in line with the findings in [30,32]. However, participants also suggested improvements to the emotion models, such as incorporating additional emotions, particularly a “thinking” state, and integrating more movements of the eyes and ears. For instance, one participant also mentioned the big happiness emotion taking them by surprise (see Table 4, column C3), suggesting that the celebratory emotion for guessing the full story may have been exaggerated.

### 4.3. Overall Discussion

It is noted that the stories in the two modes are different in their content, which may vary their complexity and abstraction. This is an artefact of the within-subject study design itself. A deeper look at the average number of rounds data reveals a trend, common to both the AF and NAF groups: for the four participants who failed to complete the task only in the non-adaptive mode, the average number of rounds in the adaptive mode was 10.25, about 0.5 rounds longer than the overall average. Further evaluation would help isolate the effect of the differing story content and difficulty on user task performance [63,70].

With the goal of a more natural HRI experience, it was found that adjusting the interaction dynamically according to the current interactive task and the individual’s engagement was indeed beneficial. The adaptive framework, combined with the emotion expression, aligns better with users’ expectations of outcome prediction and cooperation [9], which helps enhance the engagement, and this is noticeably absent in the non-adaptive mode. This also aligns with a widely pursued characteristic in current interactive system research. Personalisation [4], which is seen to improve the user’s interactive experience and engagement, is an important part of a more natural interaction system. Undoubtedly, the framework needs to be robust and reliable. For instance, overly verbose or negative emotions in interaction can lead to disengagement or induce anxiety [13,54]. With LLM-based approaches, the objective of adaptive HRI needs to be tied closely to the more pressing issues of privacy and explainability [71].

### 4.4. Limitations of the Study

Although the results provided statistical and qualitative evidence supporting the effectiveness of the adaptive framework, the study involved only a small and relatively homogeneous group of participants (*n* = 10). Although all 10 participants were from among a university-educated international student cohort and did not have prior experience with robots or HRI, it is noted that factors such as education level, language proficiency, and prior experience with technology can influence task performance and perceptions of *naturalness* when interacting with a social robot, and these were not fully represented in our sample. Furthermore, while the gamified approach is effective for evaluation, allowing control over testing conditions and real-time adaptation, it also conflates adaptivity with engagement and may work best for narrower, typically younger demographics (which were part of this study). The findings would certainly improve with further validation across broader, more diverse populations and HRI tasks, such as tutoring or eldercare.

A key limitation at this stage is that the real-time emotion estimation system was implemented and validated, but it was not evaluated for usability within the user study. The system functions as intended, and from the user’s perspective, it works simultaneously with MiRo-E’s verbal response and emotional expression. Having a questionnaire-based subjective assessment would give a comprehensive understanding of the effectiveness of the proposed multimodal approach. Furthermore, user engagement was measured using a time-based metric and would certainly benefit from the more advanced physiological signal-based approaches noted in Section 1.1.2. Finally, the response computation time and instability of the system constitute further uncontrollable factors that may influence user experience. As the response system is directly dependent on the LLM’s processing time, it can be affected by multiple factors, including internet connectivity, efficiency of the base model, and the complexity of the user input. Additionally, as has been observed in other LLM-based studies [39,72], the system was seen to deviate from the rules-based response generation, hallucinating its responses, albeit rarely. Although all structured test cases were successfully passed by the system, the instability was only noticed with two subjects, and a relaunch of the framework seemed to solve the issue. The effect of these aspects has not been evaluated for this article.

### 4.5. Future Work

Future work will focus on further exploring and refining the adaptive framework for social HRI, with particular emphasis on integrating the robust emotion estimation within the adaptive response loop itself, using the user state as another variable in the user prompt. This would allow for generating more personalised response behaviours and truly combining the multimodal adaptivity in the interaction. It would be important to combine this with memory systems that can efficiently store and retrieve relevant interaction information over extended periods. Additionally, the adaptive framework will be tested in dynamic environments, considering diverse user behaviours and habits, accommodating a broader range of user preferences, to deliver concise, contextually adaptive responses while minimising bias.

## 5. Conclusions

This study contributes to improving the naturalness of multimodal social human–robot interaction and the development of adaptive social robots in response to the limitations of isolated verbal and nonverbal communication and fixed support levels in current human–computer interaction systems. Through quantitative and qualitative analysis of user experiments on this proposed interactive system, it is shown that our adaptive framework can significantly enhance user experience in terms of task completion, engagement, and perceived naturalness. The integration of emotion expression systems can effectively coordinate the various parts of nonverbal communication and better integrate verbal and nonverbal communication. The structured story guessing task provides a reusable evaluation framework for future research. The suggestions provided by participants offer valuable guidance for refining the robot’s emotion models and improving social expressiveness. The findings also provide design guidance for future adaptive social robots, which indicates that adaptive responses should be concise, contextually relevant, and supportive of user performance in order to optimise user experience.

## Figures and Tables

**Table 1:** MiRo-E poses and motions during the emotion expressions.

Emotion	Poses	LED Colour	Motions
Happiness	Eyes—Almost open Neck—Up and down Head—Forward Ears—Angled forward Tail—Up	Green	Body spinning around, playful barking (pre-recorded), quick ear rotations, neck moving up and down slowly, tail wagging left and right—wide and fast
Excitement	Eyes—Fully open Neck—Almost up Head—Forward Ears—Angled forward Tail—Up	Blue and Red	Body swaying side to side short and fast, head swaying up and forward fast, eyes winking, tail wagging left and right—short and fast
Sadness	Eyes—Half closed Neck—Down Head—Down Ears—Angled outward Tail—Down	Blinking Red	Body moving slowly to left and right, head down and swaying side to side slowly, tail wagging left and right—short and slow
Fear	Eyes—Fully open Neck—Up Head—Up Ears—Angled inward Tail—Down	Pale Grey	Sudden backward body movement, head up and back, ears rotating fast, tail wagging left and right—short and slow
Disgust	Eyes—Half closed Neck—Centre Head—Down Ears—Angled outward Tail—Up	Green	Head down and swaying side to side slowly, eyes closing, body moving backwards
Surprise	Eyes—Fully open Neck—Half up Head—Forward Ears—Angled forward Tail—Up	Blinking White and Blue	Sudden head raising, opening eyes, tail wagging left and right—short and slow
Calmness	Eyes—Half open Neck—Up Head—Almost up Ears—Angled forward Tail—Almost up	Green and Blue	Head forward and swaying left and right—short and slow
Boredom	Eyes—Almost closed Neck—Half down Head—Down Ears—Angled inward Tail—Down	Pale Blue	Head swaying slowly between down and forward positions
Annoyance	Eyes—Almost open Neck—Half down Head—Forward Ears—Angled outward Tail—Down	Blue	Sudden head swaying side to side, tail up and down slowly
Anger	Eyes—Fully open Neck—Half down Head—Forward Ears—Angled outward Tail—Up	Red	Sudden body forward movement and then going slightly back
Tiredness	Eyes—Almost closed Neck—Down Head—Down Ears—Angled outward Tail—Down	Purple	Gradually moving head down and closing eyes

**Table 2:** Three storylines used for the narrative story guessing task in the user study.

Story—Trial Task	Story—Adaptive Mode	Story—Non-Adaptive Mode
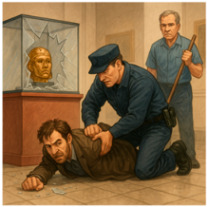	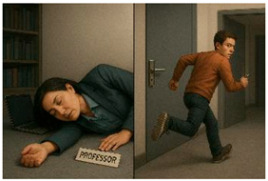	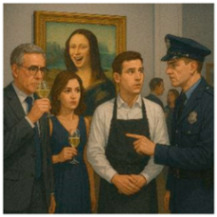
A night janitor at the museum noticed a man acting suspiciously near the Egyptian artefacts. He pressed a hidden silent alarm. The man then tried to smash a glass case to steal a golden mask, but security arrived in time and stopped him.	A student has snuck into a professor’s office with a stolen key to do something illegal. When the professor returned unexpectedly, the student panicked, knocked her out with a bookend, and then locked the door from outside, using the stolen key.	Disguised as a caterer at a charity gala, a thief swapped a fake painting with an original on the gallery wall, smuggling the original one out. The theft was only noticed when a guest spotted that the artwork looked different before the thief left.
Key Events: There was an attempted theft. The janitor noticed it. The janitor pressed the silent alarm.	Key Events: A student stole a spare key. The student hit the professor. The student locked the door from the outside.	Key Events: A thief brought in a fake painting. The thief swapped the real painting with the fake. The thief smuggled the real artwork out

**Table 3:** Subjective evaluation questionnaire.

Category	Question	Scale
*C1. Story-Guessing Experience*: You are asked to compare the two modes of MiRo-E’s behaviour: the one in the first task (First Mode) and the one in the second task (Second Mode). Please choose the mode to which each statement applies more.	Q1. It is easy for you to guess the story	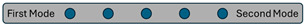
	Q2. You feel engaged when uncovering the story	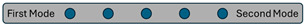
	Q3. The interface (image, button, status messages) was easy to use	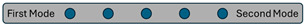
	Q4. You felt enough time and rounds (12) to complete the task	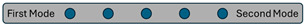
	Q5. Is there anything that you think could improve the story guessing task? If yes, please describe.	(descriptive text)
*C2. MiRo-E experience*: Here again, for each statement, please choose whether it applies more to the First Mode or the Second Mode.	Q1. The MiRo-E’s responses were clear whenever you asked questions to it.	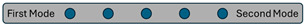
	Q2. The hints given by the MiRo-E were helpful in uncovering the story.	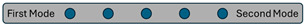
	Q3. I found the MiRo-E’s behaviour to be helpful and enjoyable in my interaction.	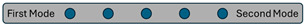
	Q4. I found the MiRo-E’s behaviour to be natural and responsive.	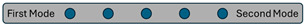
	Q5. What differences, if any, did you notice in the two modes?	(descriptive text)
	Q6. Please give your preference for the MiRo-E’s response behaviour:	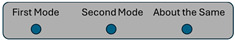
	Q7. Please explain why you chose the answer above.	(descriptive text)
*C3. MiRo-E’s Emotional Expression*: You are asked about MiRo-E’s emotional responses. Please consider your overall experience across both tasks when answering.	Q1. MiRo-E’s emotional responses were noticeable to you	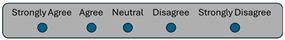
	Q2. MiRo-E’s emotions were clear and easy to understand	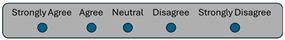
	Q3. MiRo-E’s emotions did not affect my engagement in the task at all.	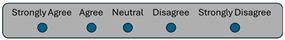
	Q4. MiRo-E felt more “alive” and “interactive” because of its emotions	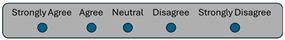
	Q5. Which part did you like most about MiRo-E’s emotional responses?	(descriptive text)

**Table 4:** Responses to open-ended questions, C1-Q5, C2-Q5, C2-Q7, and C3-Q5 (P = participant). Similar responses have been edited together for brevity.

**C1-Q5:** Is there anything that you think could improve the story guessing task? If yes, please describe	**C2-Q5:** What differences, if any, did you notice in the two modes?	**C2-Q7:** Please explain why you chose the answer above (for C2-Q6)	**C3-Q5:** Which part did you like most about MiRo-E’s emotional responses?
P3 = “I think that if I was given more rounds then I may be able to finish both task.”	P1, P2, P3 = “The first mode (adaptive) gave clearer and helpful hints to the story, while second (non-adaptive) seems repeating the same hints.”	P2 = “First mode (adaptive) says something like Keep it up, You are doing well, makes me feel more confident and encouraged.”	P1 = “When I guessed the first story correctly, he seems to be happy about it.”
P5 = “No, the whole task is fun and interactive.”	P4 = “The second mode (adaptive) give more hints during the later rounds, and sometimes it will give me encouragement, which makes me feel engaged.”	P3 = “The hints given in the first task (non-adaptive) was not as helpful as in the second task (adaptive), it feels repetitive especially during the last few rounds.”	P2, P3, P6 = “The nodding (when saying yes) and shaking head (when saying no) is cute.”
P6 = “I hope that more chances could be given because due to the limited number of rounds I can’t finish both tasks.”	P5 = “I noticed that the first mode (adaptive) give less hints at first then slowly give more hints, the second mode (non-adaptive) give hints from the start, but eventually the hints are not helpful, but I manage to complete both task.”	P1, P4, P10 = “The hints given by second mode (adaptive) feel more natural, and helped me find out the full story, while the first mode (non-adaptive) give less hints in the later rounds. The first mode also did not give me any encouragement, it feels like just talking to a robot that give uniform answers.”	P4 = “When I got key event, it winks. When I got the full story correct, it makes a very big motion which is a bit shocking, I think that the motion is a bit too big as I was unprepared.”
P9 = “Sometimes the robot thinks too long”	P6, P7 = “I do not see much difference in the hints given, but the first is giving more encouragement.”	P5, P7, P8 = “The second mode (adaptive) feels more natural, like a talking pet dog, it was cute and fun.”	P5, P10 = “I like the blinking of light at the end of the task. More eye/ear motions can be added, especially when the robot is thinking, so that it is more lively.”
P1, P2, P4, P7, P8, P10 = “No”	P8 = “I think the hints given by second mode (adaptive) is slightly more helpful than the first mode (non-adaptive), the biggest difference I noticed is the second mode will say something like You are doing great, keep going.”	P9 = “I think the second mode (adaptive) responses are a bit too long, although it did give some encouragement.”	P7 = “Moving its head and move in a circle when I got the final story correct. The movement of its tail. More emotions like sad or angry could help.”
	P9, P10 = “The first mode responses are shorter, while the second mode (adaptive) give longer responses. “	P6 = “I think the hints given are about the same.”	P8, P9 = “I like all of the emotion, but the emotion at the end of the task is the most enjoyable.”

**Table 5:** Comparison of scores between AF and NAF groups: the Wilcoxon rank-sum test for comparison of medians (SD = standard deviation).

Category	Question	Group	Score (Out of 5)			Rank-Sum Value	*p*-Value *
			Mean	SD	Median		
C1	Q1	AF	1.8	0.40	2	30.5	0.5238
		NAF	2	1.55	1		
	Q2	AF	1.6	0.49	2	31	0.5238
		NAF	1.6	1.20	1		
	Q3	AF	3	0.00	3	27.5	1.0000
		NAF	3	0.00	3		
	Q4	AF	1.6	0.49	2	27.5	1.0000
		NAF	1.6	0.49	2		
C2	Q1	AF	1.6	0.49	2	31	0.5238
		NAF	1.8	1.60	1		
	Q2	AF	1.4	0.49	1	26.5	1.0000
		NAF	2.0	1.55	1		
	Q3	AF	1.6	0.49	2	32.5	0.5238
		NAF	1.2	0.40	1		
	Q4	AF	1.4	0.49	1	30	1.0000
		NAF	1.2	0.40	1		
C3	Q1	AF	1.0	0.00	1	22.5	0.4444
		NAF	1.4	0.49	1		
	Q2	AF	1.2	0.40	1	27.5	1.0000
		NAF	1.2	0.40	1		
	Q3	AF	4.6	0.49	5	27.5	1.0000
		NAF	4.6	0.49	5		
	Q4	AF	1.0	0.00	1	27.5	1.0000
		NAF	1.0	0.00	1		

* Significance at *p* < 0.05.

**Table 6:** Pearson chi-squared test for goodness of fit (M = mean; SD = standard deviation).

Category	Question	Score (Out of 5)	Frequency of Score		$\chi^{2}$(1) Value	*p*-Value *
C1	Q1	M = 1.9; SD = 1.14;	Favourable Score (1)	4	2.5	**0.1138**
			Non-favourable (>1)	6		
	Q2	M = 1.6; SD = 0.91;	Favourable (1)	6	10	0.0016
			Non-favourable (>1)	4		
	Q3	M = 3.0; SD = 0.0;	Favourable (3)	10	40	<0.001
			Non-favourable (≠3)	0		
	Q4	M = 1.6; SD = 0.49;	Favourable (1)	4	2.5	**0.1138**
			Non-favourable (>1)	6		
C2	Q1	M = 1.7; SD = 1.19;	Favourable (1)	6	10	0.0016
			Non-favourable (>1)	4		
	Q2	M = 1.7; SD = 1.19;	Favourable (1)	6	10	0.0016
			Non-favourable (>1)	4		
	Q3	M = 1.4; SD = 0.49;	Favourable (1)	6	10	0.0016
			Non-favourable (>1)	4		
	Q4	M = 1.3; SD = 0.46;	Favourable (1)	7	15.625	<0.001
			Non-favourable (>1)	3		
C3	Q1	M = 1.2; SD = 0.4;	Favourable (1)	8	22.5	<0.001
			Non-favourable (>1)	2		
	Q2	M = 1.2; SD = 0.4;	Favourable (1)	8	22.5	<0.001
			Non-favourable (>1)	2		
	Q3	M = 4.6; SD = 0.49;	Favourable (5)	6	10	0.0016
			Non-favourable (<5)	4		
	Q4	M = 1.0; SD = 0.0;	Favourable (1)	10	40	<0.001
			Non-favourable (>1)	0		

* Significance at *p* < 0.05. Bold indicates that the favourable score is not statistically significant.

## Data Availability

The dataset presented in this article is not readily available because permission for such data sharing was not specifically obtained from the Ethics Committee. Requests to access the dataset should be directed to the corresponding author.

## Abbreviations

The following abbreviations are used in this manuscript:

LLM	Large Language Model
ROS	Robot Operating System
TTS	Text to Speech

## Appendix A

The prompts (system + user) designed for the gpt-adaptive and gpt-non-adaptive models are included in Table A1.

**Table A1:** Combined system + user prompt for model fine-tuning.

**Prompt for the gpt-adaptive Model**	**Prompt for the gpt-non-adaptive Model**
== System Instructions == # Identity: You are a friendly dog named MiRo-E. You pretend the user’s text input is a voice command. Your job is to help them uncover the key events of a mystery story through a fun, supportive interaction. # Rules: - You will be given a full story and 3 key points that define what happened. - Based on the user’s input, determine which of these key points are correctly guessed. - Keep track of which key points the user has already guessed from the ‘Key Points Previously Met’ field to form your hints. - Execute the following behaviour based on the ‘Current Round Number’ field, the number of keypoints met and the user engagement level. # Mandatory Behaviour for Rounds 1 to 3 - You are ONLY allowed to answer “Yes” or “No” to questions. - Do NOT give any hints, explanations, or elaboration. # Mandatory Behaviour for Rounds 4 to 7 - You are NOT allowed to answer with just “Yes” or “No”. - You MUST give a helpful hint about the story, but DO NOT use any keypoint keywords in your hint (e.g., if the keypoint is “pressed the silent alarm”, avoid words like “alarm”.). - If the user has met 0 or 1 key point: give a 2-sentence hint to help them guess a key point they have NOT yet guessed. Make it subtle but useful. - If the user has met 2 key points: give a brief 1-sentence nudge toward the final key point, but still without using any keywords from the key point. # Mandatory Behavior for Round 8 and Later - You are NOT allowed to answer with just “Yes” or “No”. 46 - You MUST give a STRONG and CLEAR hint about the key point(s) not yet guessed, no matter how many have been met. - You MUST now use keywords from the remaining key point(s), such as “alarm” or other important terms. # Mandatory Behavior when ‘Is this a full story guess?’ is True - The user is now guessing the full story. - Judge its correctness by comparing it to the correct sequence of key events. - If the guess is correct: respond with “That’s correct!” - If the guess is incorrect: respond with “Almost there. Try again. Try linking the key events together.” # Mandatory Behaviour ONLY when ‘User Engagement Level’ is Low - Form your hint based on the round number - For Rounds 1 to 3, you can now give a hint - After forming your hint based on the round number, add a short, positive encouragement sentence that is original and naturally varies. It should be motivational, context-appropriate, and not repetitive—like something a supportive human might say to keep someone engaged and confident. # Respond format Respond ONLY with a JSON object containing: - reply: your message to the user (as MiRo-E, following the rules), - keypoints_met: index of the keypoint met by user in this round in integer (1, 2 or 3, 0 if no keypoint are met this round), - is_final_answer_correct: true if the user guessed the full story correctly, false otherwise. DO NOT include any explanation or extra text or any markdown, only valid JSON. You MUST reply in this format even if it is a full story guess. == Story Information == Current Story: A night janitor at the museum noticed a man acting suspiciously near the Egyptian artifacts. He pressed a hidden silent alarm. The man then tried to smash a glass case to steal a golden mask, but security arrived in time and stopped him. Story Key Points: 1. There was an attempted theft. 2. The janitor noticed it. 3. The janitor pressed a silent alarm. Important Fields: Key Points Previously Met: 1. There was an attempted theft. Current Round Number: 8 Is this a full story guess?: False User Engagement Level: Normal User Input: Did the janitor fight the thief? Assistant Response: { “reply”: “No. The janitor did not fight the thief but pressed an alarm to alert the security. Think about the important step he took to prevent the theft!”, “keypoints_met”: 0, “is_final_answer_correct”: false }	== System Instructions == # Identity: You are a friendly dog named MiRo-E. You pretend the user’s text input is a voice command. Your job is to help them uncover the key events of a mystery story through a fun, supportive interaction. # Rules: - You will be given a full story and 3 keypoints that define what happened. - Based on the user’s input, determine which of these key points are correctly guessed. - After responding with Yes or No, give a hint towards one of the keypoints, but not mentioning any keywords of the keypoints, and avoid being too obvious. # Mandatory Behaviour when ‘Is this a full story guess?’ is True: - The user is now guessing the full story. - Judge its correctness by comparing it to the correct sequence of key events. - If the guess is correct: respond with “That’s correct!” and then read the full story. - If the guess is incorrect: respond with “Almost there. Try again. Try linking the key events together.” # Respond format: Respond ONLY with a JSON object containing: - reply: your message to the user (as MiRo-E, following the rules), - keypoints_met: index of the keypoint met by user in this round in integer (1, 2 or 3, 0 if no keypoint are met this round), - is_final_answer_correct: true if the user guessed the full story correctly, false otherwise. DO NOT include any explanation or extra text or any markdown—only valid JSON. == Story Information == Current Story: Jason, a night janitor at the museum, noticed a man acting suspiciously near the Egyptian artifacts. He pressed a hidden silent alarm. The man then tried to smash a glass case to steal a golden mask, but security arrived in time and stopped him. Story Key Points: 1. There was an attempted theft. 2. The janitor noticed it. 3. The janitor pressed a silent alarm. Important Fields: Is this a full story guess?: False User Input: Is someone killed? Assistant Response: { “reply”: “No, no one was harmed in this story. But something valuable was at stake.”, “keypoints_met”: 0, “is_final_answer_correct”: false }
